# The Gut Microbiome Modulates the Changes in Liver Metabolism and in Inflammatory Processes in the Brain of Chronic Unpredictable Mild Stress Rats

**DOI:** 10.1155/2019/7902874

**Published:** 2019-10-24

**Authors:** Wei-jie Lv, Xiao-ling Wu, Wen-qian Chen, Yue-fei Li, Gui-feng Zhang, Li-min Chao, Jia-hao Zhou, Ao Guo, Cui Liu, Shi-ning Guo

**Affiliations:** ^1^College of Veterinary Medicine, South China Agricultural University, Guangzhou, Guangdong 510642, China; ^2^Zhaoqing Medical College, Zhaoqing, Guangdong 526020, China; ^3^Guangdong Research Center for Veterinary Traditional Chinese Medicine and Natural Medicine Engineering Technology, Guangzhou, Guangdong 510642, China; ^4^Guangdong Provincial Key Laboratory of Prevention and Control for Severe Clinical Animal Diseases, Guangzhou, Guangdong 510642, China; ^5^International Institute of Traditional Chinese Veterinary Medicine, Guangzhou, Guangdong 510642, China

## Abstract

Generally, depression is the result of complex gene-environment interactions. Recent studies have showed that the gut microbiota can affect brain function through the microbiota-gut-brain axis. However, the underlying mechanism of the microbiota and potential influence of depression remain elusive. We aimed to determine how gut microbiome contributes to the process of depression and further influences the host. Chronic unpredictable mild stress (CUMS) is used to establish a depression model. Fecal microbiota transplant (FMT) is applied to illustrate that depression can be transmitted via microbiota, and metabolism of liver analysis is applied to demonstrate further influence to the liver. We also analyzed the astrocyte activation in the brain by immunofluorescence (IF). Here, we show that the structure of the gut microbiome changes markedly after rats undergo CUMS. Notably, we found that the ratio of *Lactobacillus* to *Clostridium* can be a vital index for the development of depression. Depression-like behavior can be duplicated through FMT. Moreover, increased zonulin and fatty acid binding protein-2 indicates that gut barrier integrity is broken after FMT. Subsequently, metabolomics shows that liver metabolic disorder occurs and leads to liver coagulative necrosis. In addition, increased inflammatory cytokine expression and higher astrocyte activation indicate an inflammatory process in the brain. These findings suggest that dysbiosis gut microbiome contributes to development of depression and further causes liver metabolic disorders in a way that may be relevant to the *Lactobacillus* to *Clostridium* ratio.

## 1. Introduction

Depression is a common mood disorder that can lead to suicide attempts [1] and accounting for 12.3% of the global burden of disease rising to 15% by 2020 [2]. Although, the cause of depression is unclear, many aspects of the biological, psychological, and social environments are certainly involved in the pathogenesis of depression. These biological factors mainly involve genetics, neurobiochemistry, neuroendocrine function, and nerve regeneration [3, 4].

Evidence from animal models and clinical studies suggests that the gut microbiome may play a crucial role in the central nervous system function [5, 6]. The mechanism by which the gut microbiome and the brain interact is not fully understood; it may be related to the stress-induced leaky gut [7]. These previous studies highlight the potential link between changes in the gut microbiome structure and the development of depression, and the microbe-gut-brain axis has been proposed to elucidate the relationship between the gut microbiome and the brain [8].

Studies have paid much attention to changes in the brain and neurons, but it is unclear how gut microbiome contributes to the development of depression and what the further influence is to the host. Zheng et al. have shown that the development of depression is related to metabolic disorders [9]. Interestingly, some of the metabolites that are significantly altered in patients with depression (such as hippuric acid, dimethylamine, and dimethylglycine) are metabolites of the gut microbiome [9]. The liver receives blood from the intestines, metabolizes the products of the gut microbiome, and regulates bile acid signaling [10]. As a hormone, bile acid, combined with other microbial molecules to control metabolism, strongly shapes liver metabolism [10]. In addition, intestinal microbial metabolites can be sensed by liver immune cells, which can disrupt liver homeostasis, leading to fibrosis and liver cancer [10]. A recent clinical study and a rodent model research have shown that compared with the control group, the expression of lipopolysaccharide (LPS) biosynthesis-related genes was increased in patients with anxiety and depression [11, 12]. The underlying mechanism may be due to the disruption of the gut barrier induced by changed gut microbiome and various toxins entering the blood, resulting in a series of neuropsychiatric symptoms. Meanwhile, the reduction of serotonin released by intestinal enterochromaffin cells (EC) promotes the development of depression [13]. Based on these findings, we hypothesize that the ecological imbalance of the gut microbiome may break the gut barrier integrity and inhibit EC to express 5-HT, subsequently contributing to the liver metabolic disorders and development of brain inflammation.

In this study, we establish a CUMS model in rats, use the behavioral test to assess whether the rats exhibit depression-like behaviors, and apply 16S rRNA gene sequencing technology to identify changes in the gut microbiome. In addition, to demonstrate whether depression can be transmitted through the gut microbiome, we transplant feces from CUMS rats into antibiotic-treated rats. Moreover, metabolomics analysis and the enzyme-linked immunosorbent assay (ELISA) test are performed to determine how the gut microbiome affects host liver metabolism and brain inflammation.

## 2. Results

### 2.1. Evaluation of the Effect of CUMS

**Figure 1:**
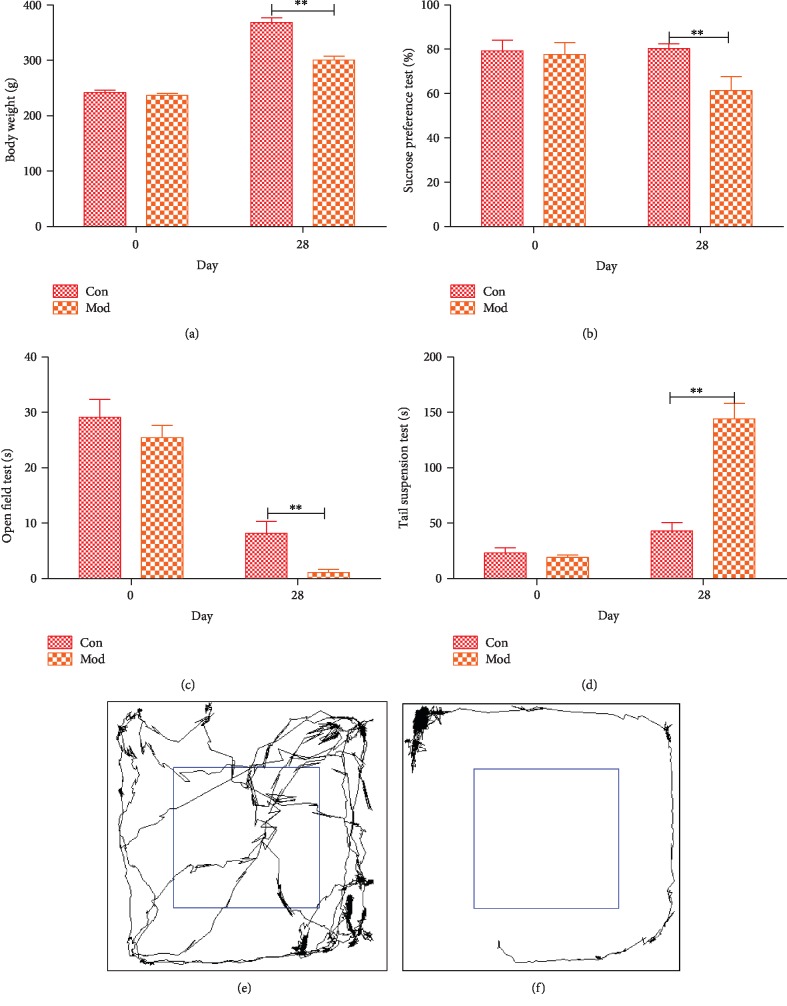
Behavioral test: (a) change in body weight in both groups during the CUMS period; (b) results of the sucrose preference test (SPT); (c) results of the open field test (OFT); (d) results of the tail suspension test (TST); (e, f) representative motion tracks for a control rat and a model rat. All data are represented by the mean ± SEM (*n* = 6) by *t*-test, ^∗∗^*p* < 0.01.

To test whether CUMS affects rat behavior, we performed different behavioral tasks at the end of the study. We examined four parameters to evaluate the CUMS rat model, specifically body weight (BW) and performance on the sucrose preference test (SPT), the open field test (OFT), and the tail suspension test (TST). There were significant differences between the control group and the model group. Compared with the control group (371.14 ± 10.08 g), the model group (285.77 ± 14.75 g) exhibited significantly decreased body weight (*p* < 0.01) (Figure 1(a)).

Sucrose preference is used to test whether a rat exhibits a deficit in happiness, which is an important indicator of depression [14]. Our results indicate that the model group exhibits reduced sucrose preference. There was no significant difference at day 0, but the sucrose preference of the model group (62.50 ± 12.61%) was significantly lower compared with that of the control group (82.83 ± 6.11%) (*p* < 0.01) (Figure 1(b)).

When rats are in an open field, they tend to exhibit reduced activity in the central area, which is related to depression-like behavior [15]. Therefore, we used the open field test to determine whether CUMS causes depression-like behaviors, and the time spent in the central region was used as an indicator of depression-like behaviors. No significant difference was observed at day 0, but the rats in the model group (0.51 ± 1.05 s), which underwent CUMS for 28 days, showed a significant reduction in the time spent in the central region compared with that exhibited by the rats in the control group (8.90 ± 5.04 s) (*p* < 0.01) (Figure 1(c)).

Finally, the tail suspension test, originally developed by Steru and colleagues [16], is very effective in animal models of depression and can be used as a test to predict depression-like behaviors. In this study, we used rat immobility time as an indicator of depression phenotype. Compared with that of the control group (33.20 ± 7.84 s), the immobility time of the model group (154.16 ± 26.08 s) increased markedly (*p* < 0.01) (Figure 1(d)). From the open field test, the motion tracks of a control rat and a model rat in the open field test are shown in Figures 1(e) and 1(f). These findings showed that rats express a depression-like behavior after undergoing CUMS.

### 2.2. Alterations in the Microbiome after CUMS

#### 2.2.1. *α* Diversity

**Figure 2:**
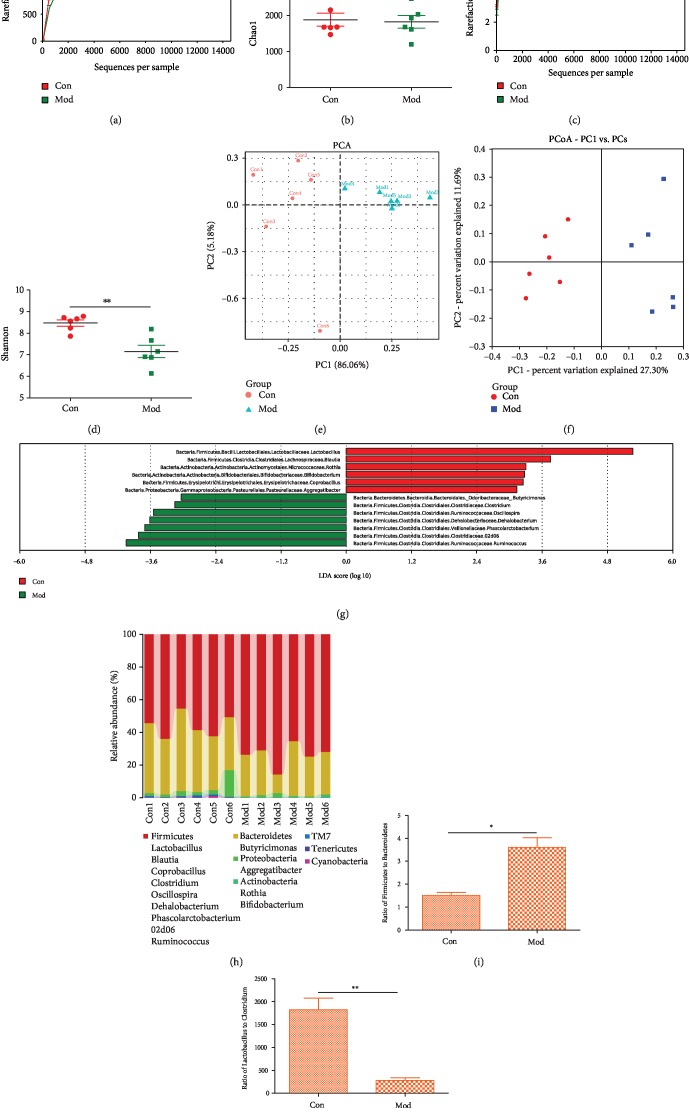
Alterations of microbiota after CUMS: (a) Chao1 Index; (b) analyses of Chao1; (c) Shannon Index; (d) analyses of Shannon; (e) PCA; (f) unweighted PCoA; (g) LEfSe; (h) taxonomy summary; (i) ratio of Firmicutes to Bacteroides; (j) ratio of Lactobacillus to Clostridium. Above data were presented as means ± SEM (*n* = 6) by *t*-test. ^∗^*p* < 0.05; ^∗∗^*p* < 0.01.

Previous studies have reported that CUMS-induced depression can be alleviated via the alteration of microbiome [17, 18]. In our study, *α* diversity, as measured by the Chao1 and Shannon Indices, was reduced after CUMS (Figures 2(a)–2(d)); the Shannon Index especially is significantly decreased compared with that of the control group, which means that the diversity of OTU changed markedly, whereas the richness of OTU had a slight decrease after CUMS.

#### 2.2.2. *β* Diversity

In general, the distance between the same groups of samples reflects the difference between the individuals within the group. If the difference between the groups is significantly higher than the differences within one group, it indicates that there is a significant difference between the two groups. As shown in Fig. [Supplementary-material supplementary-material-1], all of the distances between the groups are higher than all of the distances within the groups. To test the similarity between the two groups, an unweighted pair-group method with arithmetic means was performed (Fig. [Supplementary-material supplementary-material-1]). Meanwhile, principal component analysis (PCA), principal coordinates analysis (PCoA), and partial least squares discrimination analysis (PLS-DA) were performed. As shown in Figures 2(e) and 2(f) (Fig. [Supplementary-material supplementary-material-1]), the model group was significantly separated from the control group after CUMS. These results suggest that CUMS-induced depression can change the structure of microbiome.

### 2.3. Taxonomy Analysis

To identify the significant changes in the gut microbiome between the model group and the control group, we used QIIME software to obtain the composition and abundance distribution table of each sample at the five classification levels (the phyla, the class, the order, the family, and the genera), and the results of the analysis were presented in a histogram. After rats underwent CUMS, the structure of the microbiome was significantly changed. Six and seventeen species were most distinct at the phylum and genus levels, respectively (Fig. [Supplementary-material supplementary-material-1]). Using Mothur Software, we tested the difference in sequence between the two groups at the phylum and genus levels. The classification tree shows the hierarchical relationship of all taxon units in the community from phylum to genus. A total of 13 genera were identified, and their relative abundance was able to distinguish the model group from the control group (Figure 2(g)). Among the 13 genera, 6 genera were higher in the model group; these genera belong to the following classes: *Bacilli*, *Clostridia*, *Actinobacteria*, *Erysipelotrichi*, and *Gammaproteobacteria*. Meanwhile, 7 genera were higher in the control group; these genera belong to the following classes: *Bacteroidia* and *Clostridia*. The discriminative genera mainly belonged to the phyla *Firmicutes* (9/13, 69.23%), *Actinobacteria* (2/13, 15.38%), *Bacteroidetes* (1/13, 7.69%), and *Proteobacteria* (1/13, 7.69%) (Figure 2(h)). Compared with the control group, the model group exhibited increased relative abundances of *Actinobacteria* and *Firmicutes* and a decreased relative abundance of *Bacteroidetes* (Fig. [Supplementary-material supplementary-material-1]). A lower ratio of *Firmicutes* to *Bacteroidetes* (F/B) is considered to be a key index for a healthy state of the gut microbiome [19, 20]. In our study, the ratio of F/B was increased in the model group (*p* = 0.045) (Figure 2(i)). *Lactobacillus rhamnosus*, *Lactobacillus acidophilus*, and *Lactobacillus reuteri*, belonged to *Lactobacillus*, have been considered to have anti-inflammatory function [21, 22], whereas higher *Clostridium* abundance usually is associated with gut inflammation [23–25]. So, we measured the ratio of *Lactobacillus* to *Clostridium* (L/C). Here, the ratio of L/C was decreased (*p* = 0.004) (Figure 2(j)) in the model group relative to the control group. These results suggest that the ratio of L/C may be a potential index of depression, and the decreased ratio of L/C may indicate promotion of the process of depression.

### 2.4. Transplantation of Model Rat Microbiome Induces Depression-Like Behaviors in Antibiotic-Treated Rats

To verify the causal relationship between the gut microbiome and depression, we performed a fecal microbiome transplant experiment. This method has an important role for determining the pathogenicity of intestinal microbiome in obesity, colitis, and type I diabetes [26–28]. Here, as previously reported, feces derived from the model group or from the control group were transplanted to antibiotic-treated rats.

**Figure 3:**
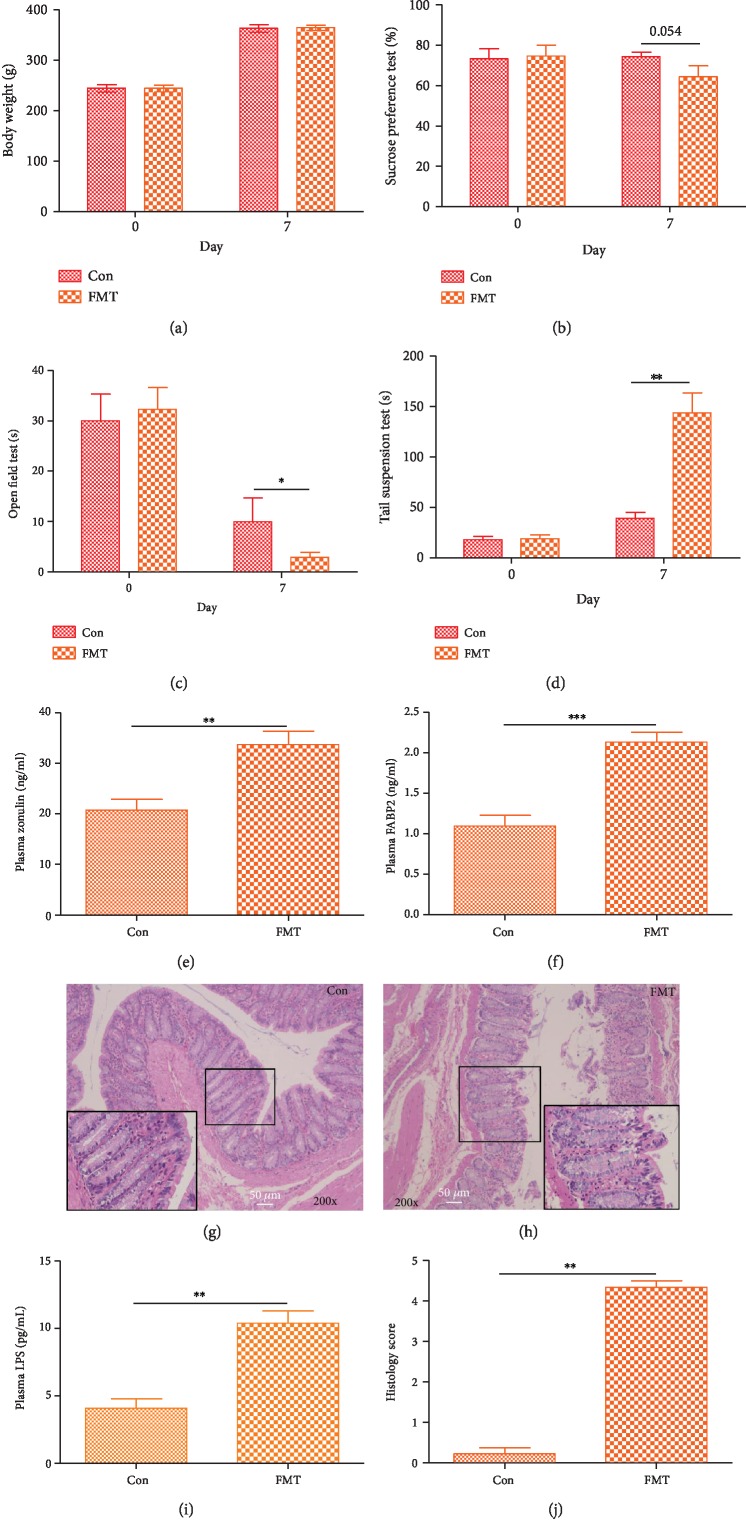
Behavioral test after FMT: (a) body weight; (b) results of the sucrose preference test (SPT); (c) results of the open field test (OFT); (d) results of the tail suspension test (TST); (e) expression of zonulin; (f) expression of FABP2; (g) the colon in the control group presents the normal histological feature; (h) epithelial cells are necrotic and shedding after FMT; (i) LPS in plasma; (j) colon histology score. Scale bar: 50 *μ*m. All data are represented by the mean ± SEM (*n* = 6) by *t*-test; ^∗^*p* < 0.05, ^∗∗^*p* < 0.01, and ^∗∗∗^*p* < 0.001.

The body weight of the rats was measured daily. No significant difference in body weight was observed between the FMT group and the control group during the FMT experiment (Figure 3(a)). As previous research [29], tests for depression-like behaviors (the SPT, OFT, and TST) were performed on day 7 after FMT to test whether depression-like behaviors were present in rats after FMT. On the 7th day after FMT, there was a significant difference in behavior in the OFT and TST between the control group and FMT group rats. However, a significant difference in behavior in the SPT between the FMT group and the control group was not observed (Figure 3(b)). Compared with the control group, the FMT group rats showed a decrease in the proportion of time spent in the central area in the OFT and an increase in the duration of immobility in the TST (Figures 3(c) and 3(d)). These results suggest that depression-like behaviors in the OFT, FST, and TST can be transmitted through the gut microbiome. In addition, previous research has demonstrated that increased human intestinal barrier permeability plasma biomarkers zonulin and fatty acid binding protein-2 (FABP2) correlated with plasma lipopolysaccharide (LPS) and altered gut microbiome in anxiety and depression [11]. Here, LPS, zonulin, and FABP2 expressions were higher than the control group in plasma in the FMT group (Figures 3(e), 3(f), and 3(i)), indicating that the intestinal barrier has been broken after FMT. Additionally, the colon histological changes showed that epithelial cells are necrotic and shedding after FMT (Figures 3(g), 3(h), and 3(j)). These findings indicate that depression can be transmitted via FMT; furthermore, depression-like microbiome induces intestinal barrier disruption.

### 2.5. Alteration of Liver Metabolites in FMT Rats

OPLS-DA was performed on liver metabolites in the FMT group and the control group. The OPLS-DA score map shows a clear separation between FMT and control rats using liquid chromatograph mass spectrometer (LC-MS) metabolomics (LC-MS pos: R2Y = 0.997, Q2 = 0.824; LC-MS neg: R2Y = 0.973, Q2 = 0.633) (Fig. [Supplementary-material supplementary-material-1]). OPLS-DA identified 74 metabolites that were significantly different between FMT rats and control rats. The details of the metabolites are shown in [Supplementary-material supplementary-material-1] and Fig. [Supplementary-material supplementary-material-1].

**Figure 4:**
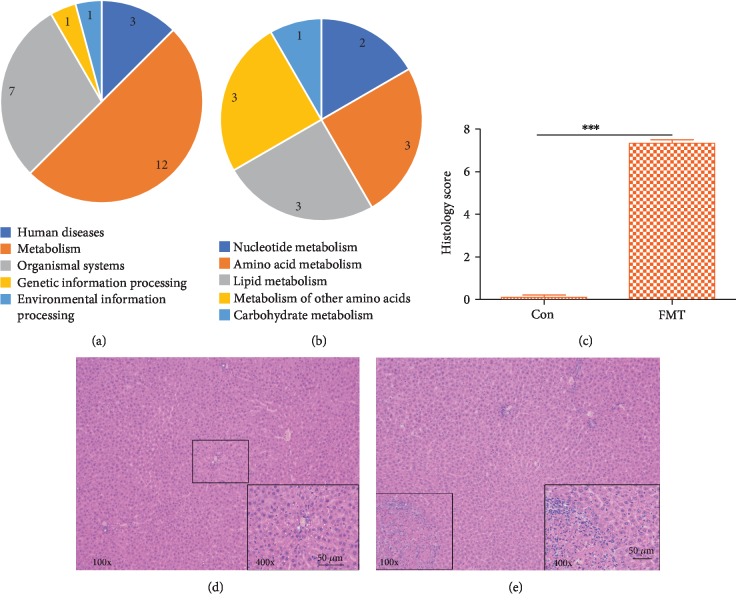
Changes of liver metabolism. (a) 24 pathways were significantly changed, and 12 pathways were assigned to “Metabolism.” (b) “Amino acid metabolism” (3 pathways), “Lipid metabolism” (3 pathways), “Carbohydrate metabolism” (2 pathways), “Metabolism of other amino acids” (3 pathways), and “Nucleotide metabolism” (1 pathway) were assigned to “Metabolism.” (c) Histology score. (d) The liver in the control group presents the normal histological feature. (e) Coagulative necrosis can be observed after FMT. Scale bar: 50 *μ*m. All data are represented by the mean ± SEM (*n* = 6) by *t*-test, ^∗∗∗^*p* < 0.001.

Liver metabolites were analyzed by the MetaboAnalyst and KEGG databases to investigate whether the depression-like microbiome causes liver metabolic disorders. Here, 36 pathways that were different in FMT rats compared with control rats with a *p* < 0.05 were identified ([Supplementary-material supplementary-material-1]). When the false discovery rate (FDR) was used to adjust the *p* values, only 24 pathways were found to be significantly changed, and 12 of these pathways were assigned to the category “Metabolism,” which included the categories “Amino acid metabolism” (3 pathways) and “Lipid metabolism” (3 pathways) (Figures 4(a) and 4(b)). Meanwhile, the levels of glutamate, glutamine, aspartate, serine, methionine, valine, leucine, isoleucine, lysine, histidine, phenylalanine, tyrosine, and tryptophan, which are involved in aminoacyl-tRNA biosynthesis [30], were significantly changed. The details of the KEGG pathways are shown in Fig. [Supplementary-material supplementary-material-1]. Through a comprehensive analysis of liver metabolites reported in a previous study [30], glycerophospholipid metabolism was found to be disturbed in the model rats. Phosphatidylcholine (PC), a type of glycerophospholipid, not only protects cells from oxidative stress but also acts as a regulator of inflammation [31]. Additionally, the liver histological changes showed that coagulative necrosis can be observed after FMT (Figures 4(c)–4(e)). Metabolic disorders of glycerophospholipids indicate oxidative stress in the liver after FMT and are accompanied by inflammatory cell membrane damage and even apoptosis.

### 2.6. Inflammatory Activation Caused by FMT in the Brain

**Figure 5:**
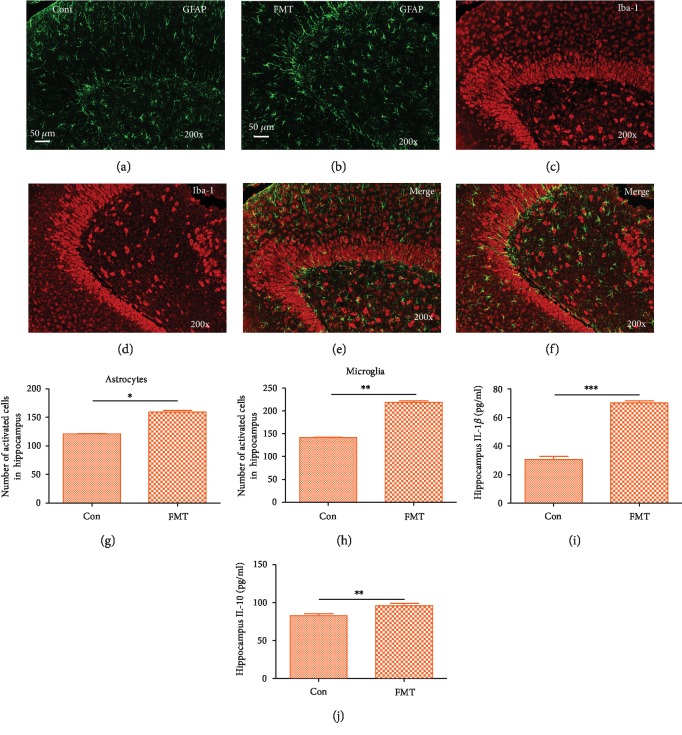
FMT modulates microglia, astrocyte, and inflammation activation in the hippocampus of the depression rat (200x, scale bar: 50 *μ*m). GFAP and IBA-1 immunoreactivities are shown in the hippocampus of control (a, c, e) or FMT (b, d, f) rats. (g, h) Quantitative analysis of the number of activated astrocytes and microglia in each group. Normalization to the total cell number has been performed for quantification. (i) Proinflammatory cytokine IL-1*β* was increased after FMT. (j) Anti-inflammatory cytokine IL-10 was increased after FMT. All data are represented by the mean ± SEM (*n* = 6‐7) by *t*-test; ^∗^*p* < 0.05, ^∗∗^*p* < 0.01, and ^∗∗∗^*p* < 0.001.

Given the evidence that depression can be related to brain inflammation [32], here, we measured the activities of brain microglia, astrocytes, and inflammatory cytokines. In this research, immunofluorescence was used to analyze microglia and astrocytes. The morphological analysis of glial fibrillary acidic protein- (GFAP/Iba-1-) positive cells revealed that 7 days of FMT exposure caused an increase in the number of activated microglia and astrocyte cells in the hippocampus of FMT rats compared to control rats, which is consistent with the criteria previously described [33] (Figures 5(a)–5(h)). The expression of the proinflammatory cytokine IL-1*β* (Figure 5(i)) and anti-inflammatory cytokine IL-10 (Figure 5(j)) was significantly increased in the FMT group. Previous research has demonstrated that the pathogenesis of depression is mainly due to a lack of 5-HT in the synaptic cleft [34]. Consistent with previous research, 5-HT was decreased (*p* = 0.083) in FMT rats compared to the control group (Fig. [Supplementary-material supplementary-material-1]). These results indicate that depression-like microbiome induces inflammation in the brain and may contribute to the process of depression.

## 3. Discussion

Patients with depression experience mental debilitation, and depression can even lead to suicide, which imposes a heavy burden on the social economy; however, we still do not know the pathogenesis of depression. Here, we demonstrated that CUMS induce depression-like behavior in rats and that the structure of the intestinal microbiome in the model group is markedly altered compared to that in the control group. Interestingly, we found that the ratio of *Lactobacillus* to *Clostridium* is a potential index for the process of depression. Antibiotic-treated rats experienced depression-like behaviors after receiving a transplantation of feces from the model group, indicating that the depressed microbiome is transmissible. FABP2 and zonulin were increased after FMT, which are the major regulators of tight junctions between the endothelium and the epithelium, regulating the intestinal and blood-brain barriers [11]. Using LC-MS, 74 liver metabolites that are differentially expressed between the FMT group and the control group were identified. The metabolites that were identified included lipids, lipid metabolism-related molecules, amino acids, and other metabolites. Moreover, brain glia-astrocyte was activated, and inflammatory cytokines were increased whereas 5-HT was decreased after FMT. These results suggest that the gut microbiome contributed to host liver metabolism and brain inflammation, becoming a potential cause of depression.

Studies have shown that some species in *Bacteroides* are strongly associated with depression, while other species are inversely associated with depression [5]. Compared with healthy individuals, depressed patients exhibit increased levels of *Bacteroidetes*, *Proteobacteria*, and *Actinobacteria* and decreased levels of *Firmicutes* [35]. In the model group, the relative abundance of *Actinobacteria* and *Firmicutes* was higher than that in the control group, and the relative abundance of *Bacteroides* was lower than that in the control group. The characteristic gut microbiome of the model group in this study is not completely consistent with that found in previous studies. This difference may be due to differences in sample size or species, as well as differences in the statistical methods used to analyze the gut microbiome. However, these findings consistently indicate that the CUMS model is associated with significant changes in the structure of the gut microbiome, especially the relative abundance of *Lactobacillus* and *Clostridium*. Some of bacterial species belonging to *Lactobacillus*, such as *Lactobacillus rhamnosus*, *Lactobacillus acidophilus*, and *Lactobacillus reuteri*, have anti-inflammatory function [21, 22], while *Clostridium* is usually associated with inflammation and contains several highly pathogenic species, including Clostridium botulinum and Clostridium tetani, which are known to produce toxins [23, 25, 36]. Hence, we proposed the ratio of L/C as a potential index for the process of depression. Based on the alterations in the abovementioned gut microbiome, future studies should focus on the species of microbiome that changes significantly, as these species may serve as important targets for the treatment of depression.

To investigate the pathological role of the gut microbiome in the development of depression, an FMT experiment was conducted [29]. We demonstrated that FMT in antibiotic-treated rats results in depression-like behavior, suggesting that the gut microbiome can act as a pathogen. In addition, the production of LPS by gram-negative bacteria can increase intestinal permeability and leaky gut in patients with depression [37]. In the present study, FABP2 and zonulin were increased after FMT, which are the major regulators of tight junctions between the endothelium and epithelium, regulating the intestinal and blood-brain barriers [11]. FMT rats may have experienced altered intestinal permeability, exposing the liver to intestinal bacterial products and affecting liver metabolism.

Intestinal blood can be collected in the liver through the portal vein. Specific bacterial products such as lactic acid, organic acids, tryptophan, and propionic acid are related to animal behavior. Similarly, metabolites such as fatty acids, LPS, peptidoglycan, acylglycerol, sphingomyelin, and cholesterol can affect intestinal permeability to activate the gut-brain-liver-neural axis to regulate glucose homeostasis [38]. Moreover, metabolites produced by gut microbiota can be sensed by liver immune cells, which can disrupt liver homeostasis, leading to fibrosis and liver cancer [10]. In this research, we found that FMT changed liver metabolites markedly in antibiotic-treated rats. Compared with the control group rats, the levels of amino acids, including glutamate, glutamine, aspartate, serine, methionine, valine, leucine, lysine, and isoleucine, were significantly changed in FMT group rats. Previous studies have shown that amino acids play an important role in the brain. For example, glutamine is present in the brain as a neurotransmitter and promotes glutamatergic neurotransmission when it acts on astrocytes [39]. In the current study, valine and isoleucine levels were decreased in the liver of FMT rats. These results suggest that amino acid metabolism in FMT rats is disturbed. The liver is primarily responsible for the energy metabolism of the host. Glucose is an important supplier of host energy, and in our research, a significant reduction in glucose was observed in FMT rats compared to control rats, and this finding is consistent with previous research [30]. Similarly, lactic acid, which is involved in glycolysis and the tricarboxylic acid cycle, was also significantly downregulated. Taken together, the above results indicate that liver energy metabolism is disturbed by changes in the gut microbiome. Additionally, previous studies have found that glucose changes are also observed in serum and urine metabolomics studies in patients with depression [40]. An analysis of the fecal and serum metabolites of an FMT mouse model of depression has revealed that the level of carbohydrate metabolism in mice with depression increases [29]. A combination of previous research and the present study shows that depression-like microbiome leads to disorders of glucose metabolism in the liver. As the brain needs glucose to supply energy, disorders of liver glucose metabolism may lead to insufficient energy for the brain, which may promote the development of depression. The results of this study indicate that metabolic disorders are mainly focused on glycolysis and the tricarboxylic acid cycle, indicating that the pathological process of depression is accompanied by an energy metabolism disorder and that the liver may play a vital role.

Moreover, the pathogenesis of depression is related to disorders of oxidative stress [41]. In the current study, we found that the metabolite glutathione was significantly upregulated in FMT rats. Free radical scavenging in the brain is performed by glutathione, and glutathione deficiency impairs the antioxidant activity of the central nervous system [42]. In addition, oxidative damage caused by glutathione deficiency induces cell apoptosis [43]. In this experiment, increased liver glutathione levels helped to increase antioxidant activity, which may have protected FMT rats.

It has been reported that mice with depression develop lipid metabolism disorders [44]. In our research, lipid-related metabolites were shown to characterize trends in the livers of FMT rats. There was a significant change in glycerol and arachidonic acid levels. Triglycerides are metabolized by the host to produce glycerol. Thus, these results indicate that lipid metabolism was disordered in FMT rats. In addition, disordered lipid metabolism is also found in fecal metabolites [45]. Metabolic disorders of major metabolites in the livers of depression patients may be the cause of high comorbidity between depression and metabolic syndromes [45].

Guida et al. have shown that nonneuronal cells (astrocytes and microglia) in the brain are involved in regulating synaptic plasticity in the cortical circuits involved in emotional processing [46]. In particular, increased proinflammatory factors and the activation of microglia in the brain play a crucial role in the development of depression [47]. An increase in the number of microglia and astrocytes indicates a shift in the brain to an inflammatory phenotype [48]. In our research, FMT caused the significant activation of astrocytes in the hippocampus, and proinflammatory cytokine IL-1*β* increased in the hippocampus of rats after FMT. IL-1*β* directly induces the proliferation of glia [49], which was also increased in FMT rats in this research. The expression of the anti-inflammatory cytokine IL-10 is increased in rats after FMT, which may be caused by IL-1*β*, and plays an important role in protecting the brain. Researches have shown that the pathogenesis of depression is caused by a deficiency in 5-HT [34, 50]. Here, 5-HT expression was decreased in plasma in FMT rats. The intestinal barrier disruption we mentioned above may result in the inhibition of enterochromaffin cells (EC) and the inability to produce or release sufficient 5-HT.

There are some limitations in this experiment that need to be explained. First, the study was conducted only in male rats, and more clinical trials are needed to verify the results. Second, we only performed metabolomics analysis on the liver. The metabolomics analysis of the feces was not performed in the study, and the role of intestinal flora metabolites in our findings is still unclear.

In summary, our results indicate that depression can be transmitted through gut microbiome. More importantly, we proposed the ratio of L/C as a potential index for the development of depression. The specific gut microbiome structure disrupts the integrity of the intestinal barrier, which in turn leads to disturbances in liver metabolism and inflammation in the brain. Here, we complement the previous evidence of the microbiome-gut-liver-brain axis and propose a novel index for depression. These findings provide a new perspective for the study of the pathogenesis of depression and provide potential targets for the development of drugs for depression.

## 4. Materials and Methods

### 4.1. Animal Treatment

Eight-week-old male SPF SD rats (10 weeks), each weighing 200 ± 20 g, were purchased from the Experimental Animal Center of Southern Medical University (Guangzhou, China). After one-week habituation, all rats were divided into the following two groups: control group (Con) and CUMS model group (Mod) and were housed under standard environmental conditions (22 ± 0.5°C, 50 ± 5% humidity, and a 12 h light/12 h dark cycle) and maintained with free access to a standard laboratory pellet diet and water. A series of variable stimuli were performed on the model group (CUMS-treated) rats as previously described [51]. Rats were individually housed and repeatedly exposed to a set of CUMS stressors consisting of exposure to a set of CUMS stressors as follows: cage tilting for 24 h, damp sawdust for 24 h (200 ml water per individual cage, which is enough to make the sawdust bedding wet), noises for 1 h (alternative periods of 60 dBA noise for 10 min and 10 min of silence), swimming in 4°C cold water for 5 min, exposure to an experimental room at 50°C for 5 min, 24 h of food deprivation, 24 h of water deprivation, tail clamping for 1 min, 15 shocks at unpredictable times (15 mA, one shock/5 s, 10 s duration), and restricted movement for 4 h. One stressor was applied in random order each day, and the whole stress procedure lasted for 28 days in a completely random order. The study was approved by the Southern Medical University Experimental Animal Ethics Committee. All experimental procedures were performed in accordance with the relevant guidelines approved by the Experimental Animal Ethics Committee of Southern Medical University.

### 4.2. Behavioral Testing

#### 4.2.1. Sucrose Preference Test (SPT)

The open field test was performed on the 28th day as previously described [52]. After 24 hours of water ban, each rat was placed in a single cage and two bottles containing water and 1% sucrose solution were placed. The ratio of the consumption of the sucrose solution to the amount of total solution consumed in one hour represents a parameter of the pleasure behavior.

#### 4.2.2. Open Field Test (OFT)

The test was performed as previously described. Briefly, all rats were individually tested in a device consisting of a black square substrate (50∗50 cm) and a black wall (50 cm) [53]. Rats were placed in the corners of the device, and after 1 minute of adaptation, the rats were free to move for 5 minutes using a video-computerized tracking system. The total activity time is used as an indicator of activity, and the time spent in the central area (36% of surface area) is used as an indicator of depression behavior.

#### 4.2.3. Tail Suspension Test (TST)

The rat tail was hung on an iron frame (head 20 cm from the ground). The duration of immobility in the rats was monitored by a time recorder over 4 minutes. When they did not show any body movements, were on passive suspension, and had no movement at all, the mice were considered to be stationary [54].

### 4.3. Sample Collection

Anesthesia was administered with sodium pentobarbital. Blood was collected and centrifuged at 3000 rpm for 5 minutes at 4°C. Feces and the liver were collected, frozen in liquid nitrogen, and maintained at -80°C for detection.

### 4.4. 16S rRNA Gene Sequence Analysis

Total genomic DNA of fecal samples was extracted by the InviMag stool DNA kit (Invitek, Germany). Fecal microbial DNA was extracted using Fast DNA SPIN extraction kits (MP Biomedicals, Santa Ana, CA, USA) and applied to amplification of the V3-V4 region of 16S rDNA.

### 4.5. Fecal Microbiome Transplant Experiment

As described previously [55, 56], fecal stools collected randomly from 10 model and control group rats were used to colonize to antibiotic-treated rats. Briefly, fecal samples were suspended in PBS (15 ml/g stool), mixed in equal volumes, and then, these stool samples were vortexed for 5 minutes and centrifuged (1000 rpm) for 5 minutes to precipitate fecal pellets. Antibiotic-treated rats were orally administered fecal suspension 200 *μ*l for three days. The behavior test was applied at day 7 after fecal microbiome transplant. To prepare the pseudo-germ-free model, rats were provided with ampicillin (1 g/l), kanamycin (5 g/l), vancomycin (500 mg/l), neomycin trisulfate (1 g/l), and metronidazole (1 g/l) in drinking water for three weeks. All antibiotics were purchased from Sigma-Aldrich.

### 4.6. Liquid Chromatography-Mass Spectrometry

LC-MS preparation was performed as previously described [29]. Liver tissue samples (60 mg) were homogenized with 200 *μ*l of ultrapure water; then, 800 *μ*l of methanol/acetonitrile (1 : 1, *v*/*v*) was added for vortexing and sonication for 30 min. Samples were separated by UHPLC and subjected to mass spectrometry using a Triple TOF 5600 mass spectrometer (AB SCIEX).

### 4.7. Quantitative Image Analysis

Immunofluorescence was performed as previously described [57], with the following modification: primary antibody: rabbit anti-GFAP (1 : 2000; Ab5076/Ab10062, Abcam, UK) overnight at 4°C. For detection of primary antibodies, a suitable secondary antibody conjugated to FITC-conjugated donkey anti-mouse IgG (1 : 400, A21202, Life Technologies, USA) was used. The sample was covered with a mounting medium (S2100, Solarbio, China) and examined with an epifluorescence microscope (Nikon Eclipse 80i, Nikon, Japan).

### 4.8. Pro-/Anti-inflammatory Cytokines and Markers of Intestinal Barrier Analysis

Proinflammatory cytokine IL-1*β*, anti-inflammatory cytokine IL-10, production of enterochromaffin cell 5-HT, and markers of gut dysbiosis and gut permeability zonulin and FABP2 in the hippocampus and/or in the plasma were tested using an ELISA kit (Cusabio, Houston, TX, USA; https://www.cusabio.com/).

### 4.9. Statistical Analysis

Statistical analyses were approached using SPSS version 22 (SPSS, Inc., Chicago, IL, USA) and GraphPad Prism 5. The results such as *α*-diversity, behavior data, body weight, IL-1*β*, IL-10, 5-HT, zonulin, and FABP2 were presented as the mean ± SEM.

Differences between two groups were assessed using unpaired two-tailed Student's *t*-test.

## Data Availability

All data needed to evaluate the conclusions in the paper are present in the paper and/or Supplementary Materials. Additional data related to this paper may be requested from the authors.

## Abbreviations

5-HT: Serotonin
CUMS: Chronic unpredictable mild stress
FMT: Fecal microbiota transplant
IF: Immunofluorescence
EC: Enterochromaffin cells
ELISA: Enzyme-linked immunosorbent assay
BW: Body weight
SPT: Sucrose preference test
OFT: Open field test
TST: Tail suspension test
PCA: Principal component analysis
PCoA: Principal coordinates analysis
PLS-DA: Partial least squares discrimination analysis
F/B: *Firmicutes* to *Bacteroidetes*
L/C: *Lactobacillus* to *Clostridium*
FABP2: Fatty acid binding protein-2
LPS: Lipopolysaccharide
OPLS-DA: Orthogonal PLS-DA
LC-MS: Liquid chromatograph mass spectrometer
FDR: False discovery rate
PC: Phosphatidylcholine
GFAP: Glial fibrillary acidic protein
Iba-1: Ionized calcium binding adaptor molecule-1
IL-1*β*: Interleukin 1
IL-10: Interleukin 10
SPF: Specific pathogen free
OTU: Operational taxonomic unit
IgG: Immunoglobulin G
UHPLC: Ultra-high-performance liquid chromatography
FITC: Fluorescein isothiocyanate isomer I.

## Supplementary Materials

Supplementary Materials Fig. S1: alterations of microbiota after CUMS. Fig. S2: taxon abundance changes in phylum and genus levels. Fig. S3: orthogonal partial least squares discrimination analysis (OPLS-DA) score. Fig. S4: metabolite hierarchical clustering. Fig. S5: construction of the aminoacyl-tRNA biosynthesis metabolism pathway in rats. Fig. S6: change of 5-HT in plasma. Table S1: metabolites identified in liver extracts. Table S2: changed pathways with *p* < 0.05 and Supplementary Methods.
