# Supplementary material for: The Gut Microbiome Modulates the Changes in Liver Metabolism and in Inflammatory Processes in the Brain of Chronic Unpredictable Mild Stress Rats
Source: Oxid Med Cell Longev. 2019 Oct 24;2019:7902874. doi: 10.1155/2019/7902874 (PMC6854967; doi:10.1155/2019/7902874)
Supplement: Supplementary Materials — Fig. S1: alterations of microbiota after CUMS. Fig. S2: taxon abundance changes in phylum and genus levels. Fig. S3: orthogonal partial least squares discrimination analysis (OPLS-DA) score. Fig. S4: metabolite hierarchical clustering. Fig. S5: construction of the aminoacyl-tRNA biosynthesis metabolism pathway in rats. Fig. S6: change of 5-HT in plasma. Table S1: metabolites identified in liver extracts. Table S2: changed pathways with p < 0.05 and Supplementary Methods. [file 7902874.f1.docx]

**Supplementary Materials**

**Fig. S1 Alterations of Microbiota after CUMS.**


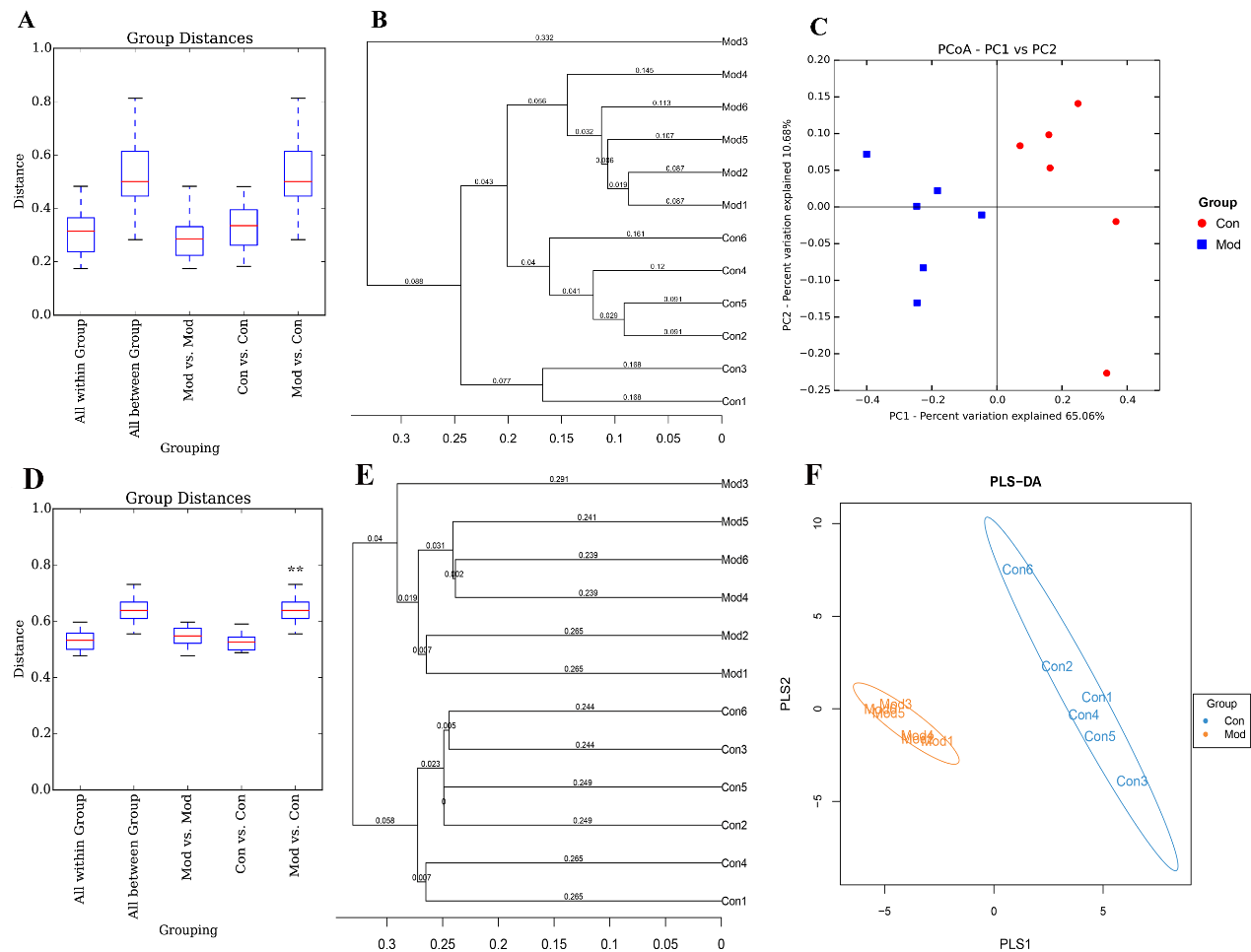


**Fig. S1** Alterations of Microbiota after CUMS. (A) Weight Group Distance. (B) Weight unifrac. (C) Weight PCoA.

**Fig. S2 Taxa abundance changes in phylum and genus level.**


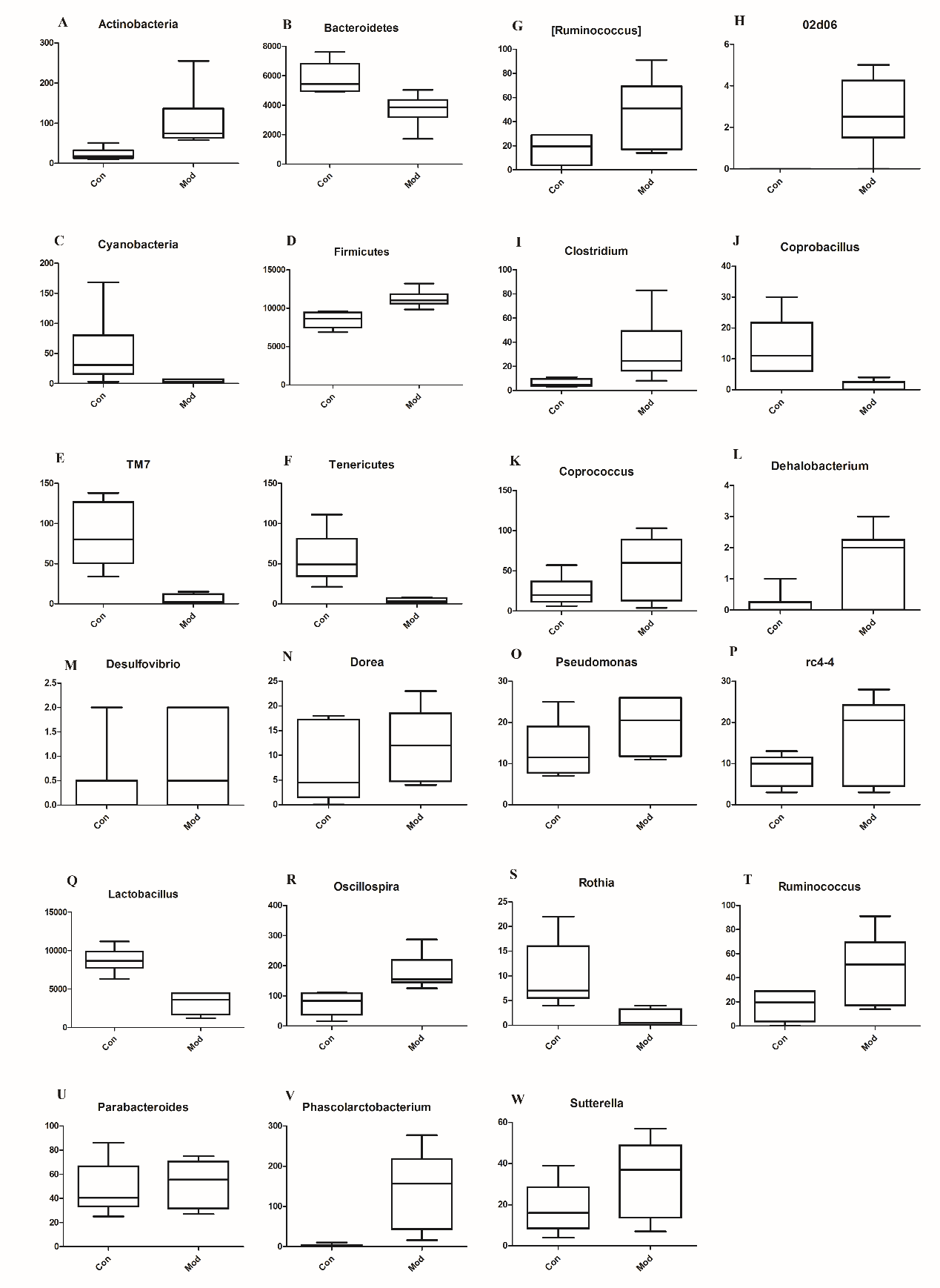


**Fig. S2** Taxa abundance changes in phylum (A-F) and genus (G-W) level.

**Fig. S3. Orthogonal partial least-squares discriminant analysis (OPLS-DA) score plots.**


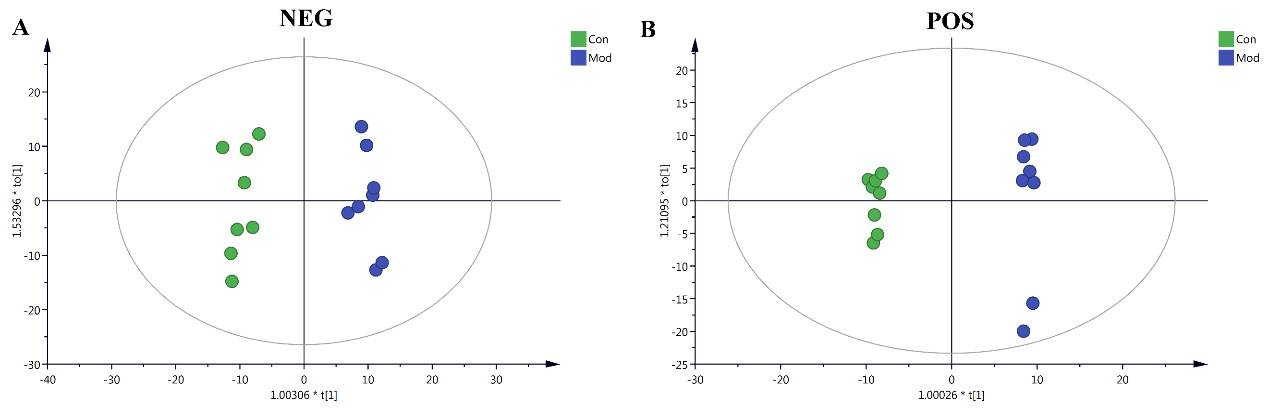


**Fig. S3**. Orthogonal partial least-squares discriminant analysis (OPLS-DA) score plots. A. B OPLS-DA score plots derived from ultra-performance liquid chromatography–tandem mass spectrometry (UPLC-Q-TOF/MS) electrospray ionization (ESI) (−), UPLC-Q-TOF/MS ESI (+).

**Fig. S4. Metabolite hierarchical clustering**


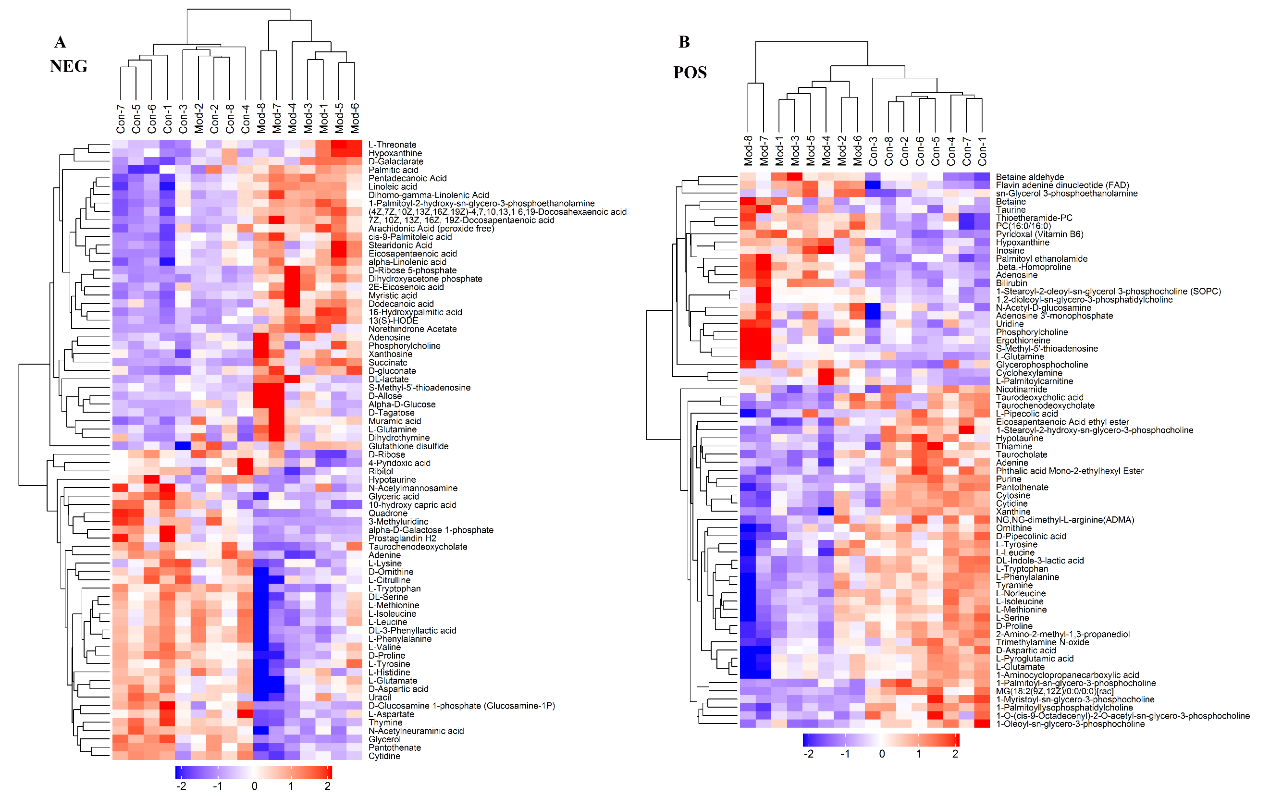


**Fig. S4**. Results of significant difference metabolite hierarchical clustering between Control and Model group.

**Fig. S5. Construction of the aminoacyl-tRNA biosynthesis metabolism pathway in rats.**

**
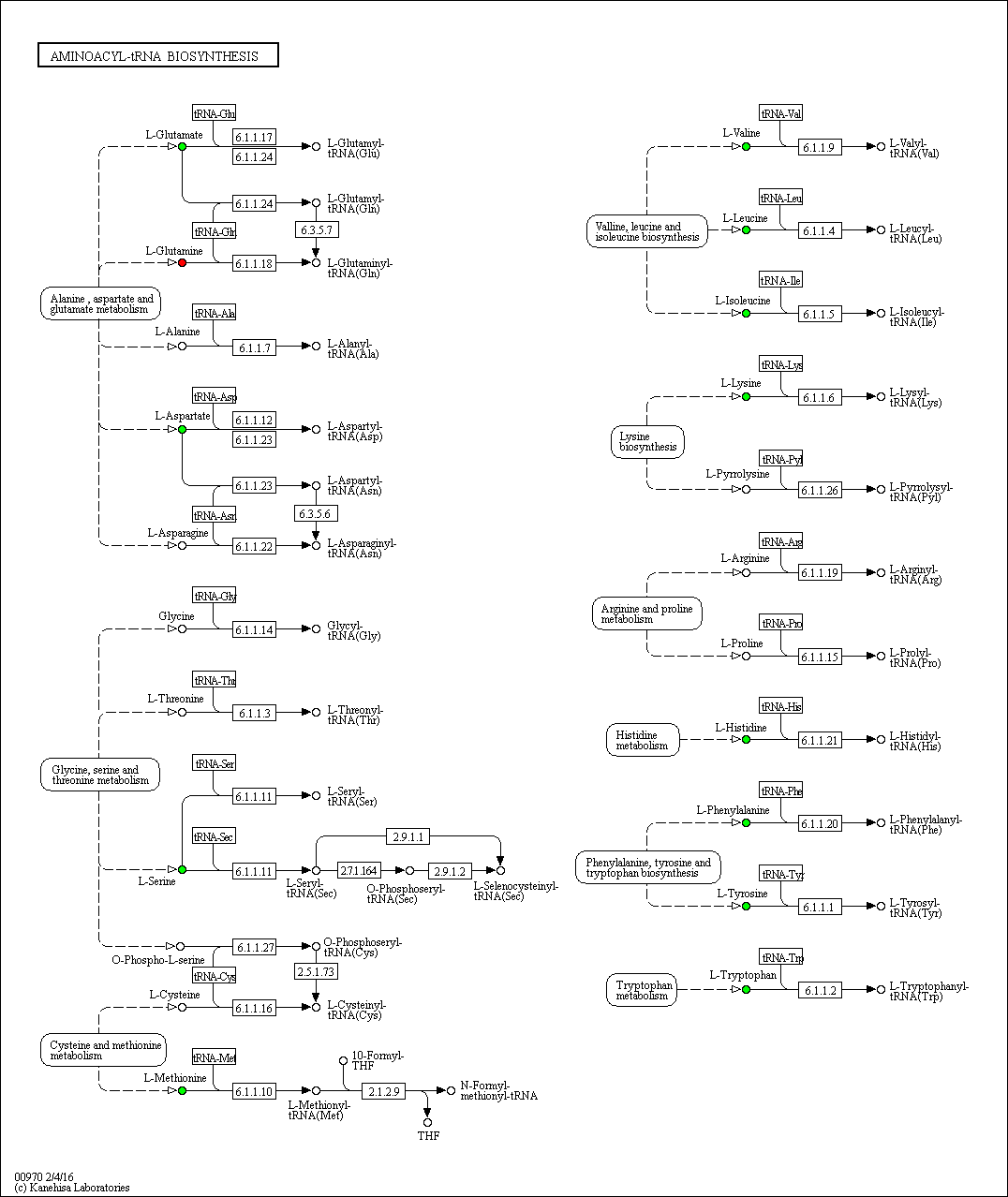
**

**Fig. S5.** Construction of the aminoacyl-tRNA biosynthesis metabolism pathway in rats. The map was generated using the reference map from Kyoto Encyclopedia of Genes and Genomes (KEGG) (<http://www.genome.jp/kegg/>). Red nodes show metabolites activation and Green nodes show metabolites inhibition.

**Fig. S6** **Change of 5-HT in plasma.**


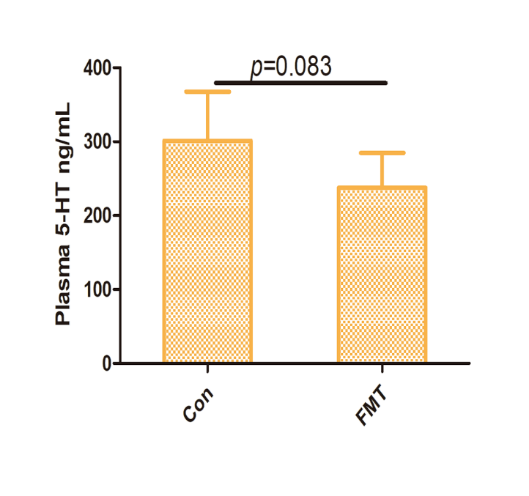


**Fig. S6** Compared with control group, 5-HT in plasma was decreased in FMT rats. (n=6)

**Table S1. Table S1 Metabolites identified in livers extracts**

**Table S1. Table S1 Metabolites identified in livers extracts**

| Metabolite/super class | VIP | Fold Change | *P*-Value |
| --- | --- | --- | --- |
| D-Glucosamine 1-phosphate (Glucosamine-1P) | 1.91587 | 0.329736 | 0.0000153 |
| Glycerol | 4.52198 | 0.62633 | 0.0000191 |
| Succinate | 1.2752 | 3.064306 | 0.0000563 |
| D-Ribose 5-phosphate | 1.96863 | 3.310335 | 0.000062 |
| Adenine | 1.21439 | 0.658512 | 0.0000808 |
| 3-Methyluridine | 1.04912 | 0.136588 | 0.00017 |
| Dihydroxyacetone phosphate | 1.49458 | 2.07452 | 0.000172 |
| L-Tryptophan | 3.4639 | 0.732905 | 0.000304 |
| 7Z, 10Z, 13Z, 16Z, 19Z-Docosapentaenoic acid | 1.63172 | 3.700313 | 0.00036 |
| Norethindrone Acetate | 2.7115 | 11.37919 | 0.000386 |
| Thymine | 1.56604 | 0.460107 | 0.00043 |
| cis-9-Palmitoleic acid | 7.37095 | 2.177963 | 0.000439 |
| 16-Hydroxypalmitic acid | 1.78992 | 2.117443 | 0.000466 |
| D-Ornithine | 1.61901 | 0.611759 | 0.000504 |
| (4Z,7Z,10Z,13Z,16Z,19Z)-4,7,10,13,1 6,19-Docosahexaenoic acid | 13.0797 | 2.102272 | 0.000714 |
| Pantothenate | 4.69544 | 0.696991 | 0.000842 |
| Pentadecanoic Acid | 1.49785 | 1.6482 | 0.000945 |
| alpha-D-Galactose 1-phosphate | 1.05149 | 0.344229 | 0.000958 |
| Muramic acid | 1.08733 | 1.726768 | 0.000991 |
| Quadrone | 1.4691 | 0.505364 | 0.001321 |
| L-Aspartate | 1.14821 | 0.670903 | 0.00181 |
| 1-Palmitoyl-2-hydroxy-sn-glycero-3-phosphoethanolamine | 1.8565 | 2.239878 | 0.001864 |
| Myristic acid | 3.74141 | 2.024779 | 0.001952 |
| Linoleic acid | 16.274 | 1.591519 | 0.002349 |
| Xanthosine | 1.71173 | 1.560811 | 0.002758 |
| N-Acetylneuraminic acid | 1.55587 | 0.839631 | 0.002895 |
| alpha-Linolenic acid | 6.36438 | 1.663611 | 0.00313 |
| Dihomo-gamma-Linolenic Acid | 3.95978 | 2.006997 | 0.003236 |
| D-Aspartic acid | 4.96278 | 0.681803 | 0.003428 |
| DL-3-Phenyllactic acid | 1.35366 | 0.743744 | 0.003742 |
| D-Proline | 4.3541 | 0.653904 | 0.003783 |
| Cytidine | 5.57282 | 0.710839 | 0.003807 |
| 13(S)-HODE | 1.53292 | 1.376227 | 0.00418 |
| Prostaglandin H2 | 1.58786 | 0.35073 | 0.004193 |
| L-Lysine | 2.4151 | 0.670849 | 0.004599 |
| Hypotaurine | 1.17015 | 0.507122 | 0.004756 |
| L-Phenylalanine | 7.25574 | 0.740331 | 0.004912 |
| D-Galactarate | 1.86234 | 1.455372 | 0.005142 |
| Eicosapentaenoic acid | 8.26149 | 2.092814 | 0.005449 |
| L-Citrulline | 1.21132 | 0.600476 | 0.00582 |
| Metabolite/super class | VIP | Fold Change | *P*-Value |
| Ribitol | 2.53826 | 0.613283 | 0.006506 |
| Dodecanoic acid | 1.07527 | 1.437298 | 0.0083 |
| D-gluconate | 1.43492 | 1.931683 | 0.009113 |
| DL-Serine | 2.29385 | 0.76475 | 0.010321 |
| Glyceric acid | 1.02489 | 0.784276 | 0.010464 |
| 2E-Eicosenoic acid | 1.30871 | 2.136134 | 0.010892 |
| L-Methionine | 3.60858 | 0.740551 | 0.012184 |
| L-Isoleucine | 1.33717 | 0.687095 | 0.017181 |
| L-Glutamine | 4.60956 | 1.900514 | 0.01787 |
| Stearidonic Acid | 1.50434 | 1.613425 | 0.019902 |
| L-Valine | 4.69697 | 0.728341 | 0.020665 |
| Taurochenodeoxycholate | 13.8418 | 0.585792 | 0.022167 |
| L-Threonate | 1.01413 | 1.502273 | 0.023096 |
| Dihydrothymine | 1.18851 | 1.782928 | 0.023158 |
| Adenosine | 2.052 | 1.89564 | 0.025617 |
| L-Glutamate | 7.81896 | 0.77457 | 0.036398 |
| 10-hydroxy capric acid | 1.02293 | 0.690689 | 0.041502 |
| DL-lactate | 4.54811 | 1.665077 | 0.045208 |
| L-Leucine | 8.65277 | 0.858907 | 0.050355 |
| N-Acetylmannosamine | 1.08891 | 0.753471 | 0.050574 |
| 4-Pyridoxic acid | 2.24733 | 0.622438 | 0.053773 |
| Glutathione | 1.08676 | 1.192196 | 0.056986 |
| Arachidonic Acid (peroxide free) | 13.0171 | 1.522829 | 0.058915 |
| Uracil | 14.5127 | 0.809772 | 0.06599 |
| D-Tagatose | 1.95352 | 1.875671 | 0.070182 |
| Palmitic acid | 1.29015 | 1.482515 | 0.071201 |
| Hypoxanthine | 3.3661 | 1.517991 | 0.074314 |
| L-Tyrosine | 4.08056 | 0.808479 | 0.07883 |
| D-Ribose | 2.11532 | 0.796977 | 0.083052 |
| D-Allose | 2.70734 | 2.30891 | 0.083131 |
| Alpha-D-Glucose | 6.45727 | 1.821837 | 0.093385 |
| Phosphorylcholine | 2.76893 | 1.671925 | 0.093856 |
| L-Histidine | 2.90109 | 0.875448 | 0.0964 |
| S-Methyl-5'-thioadenosine | 1.54382 | 2.158603 | 0.098865 |

**Table S2. Changed pathways with *P* < 0.05**

**Table S2. Changed pathways with *P* < 0.05**

| **Map. Name** | **Test** | **Ref** | ***P* value** | **FDR** | **Rich Factor** |
| --- | --- | --- | --- | --- | --- |
| Central carbon metabolism in cancer | 13 | 37 | 3.32E-12 | 3.44E-10 | 0.35 |
| Protein digestion and absorption | 14 | 47 | 6.08E-12 | 3.44E-10 | 0.30 |
| Aminoacyl-tRNA biosynthesis | 13 | 52 | 4.27E-10 | 1.61E-08 | 0.25 |
| ABC transporters | 17 | 128 | 2.43E-08 | 6.86E-07 | 0.13 |
| Mineral absorption | 8 | 29 | 5.36E-07 | 1.21E-05 | 0.28 |
| Retrograde endocannabinoid signaling | 6 | 19 | 6.58E-06 | 0.00012 | 0.32 |
| Choline metabolism in cancer | 4 | 11 | 0.00014 | 0.00224 | 0.36 |
| Purine metabolism | 9 | 92 | 0.00068 | 0.00946 | 0.10 |
| Alanine, aspartate and glutamate metabolism | 5 | 28 | 0.00075 | 0.00946 | 0.18 |
| GABAergic synapse | 3 | 9 | 0.00140 | 0.01581 | 0.33 |
| Glycine, serine and threonine metabolism | 6 | 50 | 0.00195 | 0.01846 | 0.12 |
| Alcoholism | 3 | 10 | 0.00196 | 0.01846 | 0.30 |
| Glycerophospholipid metabolism | 6 | 52 | 0.00239 | 0.01983 | 0.12 |
| Taurine and hypotaurine metabolism | 4 | 22 | 0.00246 | 0.01983 | 0.18 |
| Biosynthesis of unsaturated fatty acids | 6 | 54 | 0.00291 | 0.02058 | 0.11 |
| Arginine biosynthesis | 4 | 23 | 0.00291 | 0.02058 | 0.17 |
| Vitamin digestion and absorption | 5 | 39 | 0.00350 | 0.02326 | 0.13 |
| Linoleic acid metabolism | 4 | 28 | 0.00608 | 0.03617 | 0.14 |
| Pantothenate and CoA biosynthesis | 4 | 28 | 0.00608 | 0.03617 | 0.14 |
| Regulation of lipolysis in adipocytes | 3 | 15 | 0.00674 | 0.03810 | 0.20 |
| Galactose metabolism | 5 | 46 | 0.00719 | 0.03868 | 0.11 |
| Pyrimidine metabolism | 6 | 66 | 0.00791 | 0.04061 | 0.09 |
| Proximal tubule bicarbonate reclamation | 3 | 17 | 0.00970 | 0.04635 | 0.18 |
| beta-Alanine metabolism | 4 | 32 | 0.00984 | 0.04635 | 0.13 |
| Pentose phosphate pathway | 4 | 35 | 0.01348 | 0.06095 | 0.11 |
| Cocaine addiction | 2 | 8 | 0.01797 | 0.07522 | 0.25 |
| Glutamatergic synapse | 2 | 8 | 0.01797 | 0.07522 | 0.25 |
| Valine, leucine and isoleucine biosynthesis | 3 | 23 | 0.02249 | 0.08583 | 0.13 |
| Long-term depression | 2 | 9 | 0.02271 | 0.08583 | 0.22 |
| Glyoxylate and dicarboxylate metabolism | 5 | 61 | 0.02279 | 0.08583 | 0.08 |
| Cysteine and methionine metabolism | 5 | 62 | 0.02427 | 0.08849 | 0.08 |
| Amphetamine addiction | 2 | 10 | 0.02789 | 0.09850 | 0.20 |
| Histidine metabolism | 4 | 47 | 0.03604 | 0.12339 | 0.09 |
| D-Glutamine and D-glutamate metabolism | 2 | 12 | 0.03952 | 0.13133 | 0.17 |
| Fatty acid biosynthesis | 4 | 50 | 0.04381 | 0.14143 | 0.08 |
| Oxytocin signaling pathway | 2 | 13 | 0.04590 | 0.14407 | 0.15 |

**Supplementary Methods**

**DNA Extraction**

Total bacterial genomic DNA samples were extracted using the Fast DNA SPIN extraction kits (MP Biomedicals, Santa Ana, CA, USA), following the manufacturer’s instructions, and stored at −20°C prior to further analysis. The quantity and quality of extracted DNAs were measured using a NanoDrop ND-1000 spectrophotometer (Thermo Fisher Scientific, Waltham, MA, USA) and agarose gel electrophoresis, respectively.

**16S rDNA Amplicon Pyrosequencing**

PCR amplification of the bacterial 16S rRNA genes V3–V4 region was performed using the forward primer 338F (5’- ACTCCTACGGGAGGCAGCA-3’) and the reverse primer 806R (5’- GGACTACHVGGGTWTCTAAT-3’). Sample-specific 7-bp barcodes were incorporated into the primers for multiplex sequencing. The PCR components contained 5 μl of Q5 reaction buffer (5×), 5 μl of Q5 High-Fidelity GC buffer (5×), 0.25 μl of Q5 High-Fidelity DNA Polymerase (5U/μl), 2 μl (2.5 mM) of dNTPs, 1 μl (10 uM) of each Forward and Reverse primer, 2 μl of DNA Template, and 8.75 μl of ddH2O. Thermal cycling consisted of initial denaturation at 98 °C for 2 min, followed by 25 cycles consisting of denaturation at 98 °C for 15 s, annealing at 55 °C for 30 s, and extension at 72 °C for 30 s, with a final extension of 5 min at 72 °C. PCR amplicons were purified with Agencourt AMPure Beads (Beckman Coulter, Indianapolis, IN) and quantified using the PicoGreen dsDNA Assay Kit (Invitrogen, Carlsbad, CA, USA). After the individual quantification step, amplicons were pooled in equal amounts, and pair-end 2300 bp sequencing was performed using the Illlumina MiSeq platform with MiSeq Reagent Kit v3 at Shanghai Personal Biotechnology Co., Ltd (Shanghai, China).

**Sequence Analysis**

The Quantitative Insights Into Microbial Ecology (QIIME, v1.8.0) pipeline was employed to process the sequencing data, as previously described Briefly, raw sequencing reads with exact matches to the barcodes were assigned to respective samples and identified as valid sequences. The low-quality sequences were filtered through following criteria: sequences that had a length of <150 bp, sequences that had average Phred scores of <20, sequences that contained ambiguous bases, and sequences that contained mononucleotide repeats of >8 bp. Paired-end reads were assembled using FLASH. After chimera detection, the remaining high-quality sequences were clustered into operational taxonomic units (OTUs) at 97% sequence identity by UCLUST (Edgar 2010). A representative sequence was selected from each OTU using default parameters. OTU taxonomic classification was conducted by BLAST searching the representative sequences set against the Greengenes Database using the best hit. An OTU table was further generated to record the abundance of each OTU in each sample and the taxonomy of these OTUs. OTUs containing less than 0.001% of total sequences across all samples were discarded. To minimize the difference of sequencing depth across samples, an averaged, rounded rarefied OTU table was generated by averaging 100 evenly resampled OTU subsets under the 90% of the minimum sequencing depth for further analysis.

**Bioinformatics and Statistical Analysis**

Sequence data analyses were mainly performed using QIIME and R packages (v3.2.0). OTU-level alpha diversity indices, such as Chao1 richness estimator, ACE metric (Abundance-based Coverage Estimator), Shannon diversity index, and Simpson index, were calculated using the OTU table in QIIME. OTU-level ranked abundance curves were generated to compare the richness and evenness of OTUs among samples. Beta diversity analysis was performed to investigate the structural variation of microbial communities across samples using UniFrac distance metrics and visualized via principal coordinate analysis (PCoA), nonmetric multidimensional scaling (NMDS) and unweighted pair-group method with arithmetic means (UPGMA) hierarchical clustering. Differences in the Unifrac distances for pairwise comparisons among groups were determined using Student’s t-test and the Monte Carlo permutation test with 1000 permutations, and visualized through the box-and-whiskers plots. Principal component analysis (PCA) was also conducted based on the genus-level compositional profiles. The significance of differentiation of microbiota structure among groups was assessed by PERMANOVA (Permutational multivariate analysis of variance) (McArdle and Anderson 2001) and ANOSIM (Analysis of similarities) using R package “vegan”. The taxonomy compositions and abundances were visualized using MEGAN and GraPhlAn. Venn diagram was generated to visualize the shared and unique OTUs among samples or groups using R package “VennDiagram”, based on the occurrence of OTUs across samples/groups regardless of their relative abundance. Taxa abundances at the phylum, class, order, family, genus and species levels were statistically compared among samples or groups by Metastats, and visualized as violin plots. LEfSe (Linear discriminant analysis effect size) was performed to detect differentially abundant taxa across groups using the default parameters. PLS-DA (Partial least squares discriminant analysis) was also introduced as a supervised model to reveal the microbiota variation among groups, using the “plsda” function in R package “mixOmics”. Random forest analysis was applied to discriminating the samples from different groups using the R package “randomForest” with 1,000 trees and all default settings. The generalization error was estimated using 10-fold cross-validation. The expected “baseline” error was also included, which was obtained by a classifier that simply predicts the most common category label. Co-occurrence analysis was performed by calculating Spearman’s rank correlations between predominant taxa. Correlations with |RHO| > 0.6 and *P* < 0.01 were visualized as co-occurrence network using Cytoscape. Microbial functions were predicted by PICRUSt (Phylogenetic investigation of communities by reconstruction of unobserved states), based on high-quality sequences.
